# Clinical outcomes of AIT in the elderly population

**DOI:** 10.1097/ACI.0000000000000925

**Published:** 2023-06-19

**Authors:** Andrzej Bożek

**Affiliations:** Clinical Department of Internal Diseases, Dermatology and Allergology, Medical University of Silesia, Katowice, Poland

**Keywords:** allergic rhinitis, elderly, immunoglobulin E, immunotherapy

## Abstract

**Purpose of review:**

This review aims to present the current knowledge on the effectiveness and safety of allergen immunotherapy (AIT) in patients over 60 years of age with inhalant allergies.

**Recent findings:**

Over the last 10 years, the problem of immunoglobulin E allergy in seniors has been noticed by many authors. At the same time, in the 1990s, trials of desensitization to selected inhalant allergens were started, obtaining evidence of the effectiveness of AIT, both with the use of sublingual immunotherapy (SLIT) and injection immunotherapy (SCIT), in patients over 60 years of age with allergic rhinitis. Such data have been confirmed for AITs for grasses, birch, and house dust mites. Currently, these patients are being monitored to assess the long-term effect of AIT. All available observations confirm the high safety of AIT in seniors.

**Summary:**

Seniors with allergic rhinitis or asthma may qualify for AIT if they do not have contraindications. These patients can experience a sustained clinical benefit even after completing AIT treatment. Studies indicate that injectable and sublingual routes of administration may be effective in this age group, provided the suspect allergen is accurately diagnosed.

## INTRODUCTION

Allergen immunotherapy (AIT) remains one of the most important treatment methodologies in contemporary allergology [1^▪^,^2^,^3^]. Its efficacy in treating allergic rhinitis, some forms of asthma, and allergies to Hymenoptera venoms and selected foods has been confirmed in many clinical randomized and nonrandomized trials, reports, and meta-analyses, [4–11]. Qualification criteria, contraindications, routes of administration, and the assessment of safety and efficacy depending on the desensitized allergens are included in many international consensuses, most importantly, in the European Academy of Allergy and Clinical Immunology (EAACI) recommendations [6,10,12,13].

AIT is administered to patients of various ages. However, the lower age limit for starting such therapy is five years. Many studies indicate that an early start may result in a stronger effect [10,12–14]. The upper age limit has not been defined due to many factors. Until recently, there was no information about the incidence of immunoglobulin E (IgE)-mediated allergies in seniors and the need for AIT treatment. Understanding the role of allergies in elderly patients is essential because the average life expectancy for a new-born has risen to 80 years in most Western European countries. It is also unclear how immune ageing impacts the efficacy of inducing immune tolerance with AIT. 

**Box 1:**
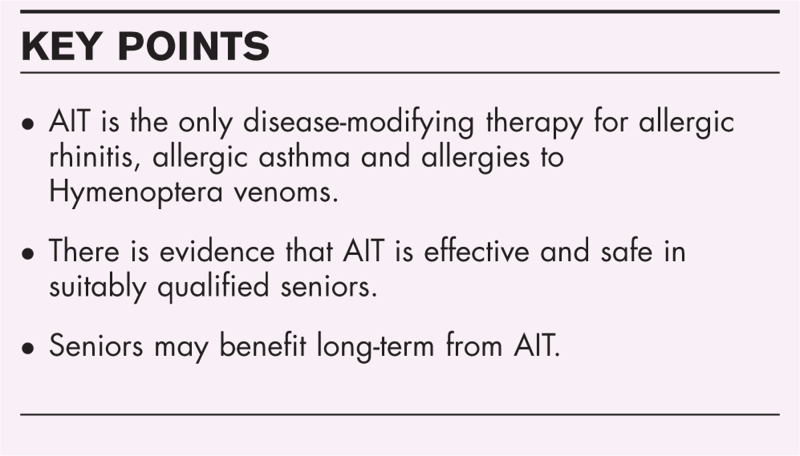
no caption available

## ALLERGEN IMMUNOTHERAPY AND IMMUNOSENESCENCE

The link between ageing and disease is partly a reflection of the functional changes in the immune system of seniors. AIT suppresses allergen-specific Th2 responses caused by increased numbers of regulatory T cells, the production of interleukin-10 and specific immunoglobulin G_4_ (IgG_4_) antibodies. AIT induces very rapid desensitization of mast cells and basophils evidenced by low responsiveness to allergens despite the high allergen specific IgE levels observed at the initiation of the therapy. Late effects of AIT are associated with a reduction of mast cell and basophil infiltration in the tissues and a reduced mediator release [15].

Immunosenescence can decrease resistance to infection, weaken responses to vaccination, and contribute to the development of age-related diseases [16^▪▪^]. Analysis of the factors contributing to this profound immune remodelling has revealed complex immune alterations that influence both the innate and adaptive arms of the immune system [16^▪▪^].

In the context of the efficacy of AIT, ageing of the immune system may reduce the ability of the immune system to respond to new antigens, promote the accumulation of memory T cells, significantly lower the number of CD4(+) and CD8(+) CD28(+) cells, induce increased numbers of CD8(+) CD28(–) apoptosis-resistant cells, and cause an inversion in the ratio of CD4/8 T cells (17,18). Overall survival is worse in patients with inversion of the CD4/CD 8 T-cell ratio, reduced proliferative response to mitogenic stimuli, and severely reduced B cell numbers. However, these quantitative changes do not appear to strongly impact the induction of allergen tolerance with AIT [17,18]. The clinical evidence for this is reviewed below.

## PREVALENCE OF IMMUNOGLOBULIN E-MEDIATED ALLERGIC DISEASES IN ELDERLY INDIVIDUALS

The incidence of allergic disease symptoms and their severity decreases with age [19,20]. However, opinions are divided as to the cause of this decline, and there is evidence that IgE-mediated disease occurs regularly in patients over 65. In the latest study of this type, Nam *et al.* determined that the prevalence of allergic diseases in the elderly is 5–10%, and the prevalence of self-reported allergic rhinitis in subjects aged >60 years was 13.0% in men and 15.4% in women [21]. Prior observations of larger groups of patients emphasized the significant occurrence of atopic diseases in elderly individuals. Although occurring less frequently than in younger patients, the incidence of atopic disease in elderly individuals points to underdiagnosis and undertreatment of elderly patients. The German ESTHER study conducted on a group of 9949 patients aged 50 to 75 confirmed the presence of bronchial asthma in 5.5% of respondents and allergic rhinitis in 8.3% [22]. Similar results were obtained in other senior populations [23,24]. Polish epidemiological data from this age group indicated the following frequencies of atopic diseases: bronchial asthma - 5.9%, atopic dermatitis - 1.6%, chronic allergic rhinitis - 17.1%, sporadic allergic rhinitis - 12.6% [25]. These data suggest that AIT treatment of seniors is controversial but necessary.

## EFFICACY AND SAFETY OF ALLERGEN IMMUNOTHERAPY IN ELDERLY INDIVIDUALS

In 1993, Armentia *et al.* confirmed that patients >60 years. showed clinical responses to injectable AIT for allergens such as mites, pollens, moulds and bee venom [26]. While this study included a placebo control group, the group of patients treated with AIT was small (14 people). Asero *et al.* confirmed the positive effect and safety of desensitization in patients over 54 years of age with periodic allergic rhinitis, asthma and allergies to birch or mugwort pollen [27]. Preseasonal injection therapy over the course of three years reduced disease symptoms by more than 50% in 95% of older patients. These rates of desensitization were comparable to a control group of younger patients (in which AIT produced an effect of more than 50% in 97% of the subjects). However, this study failed to meet the randomization and double-blind criteria [27].

Additional evidence supports the efficacy of AIT in allergic rhinitis and sensitization to allergens such as house dust mites (HDM), grasses and birch. Two studies meeting the double-blind and randomized criteria examined a specific sublingual method of immunotherapy (SLIT). The first study demonstrated efficacy and safety of SLIT in patients over 60 years of age who were allergic to HDM and with perennial allergic rhinitis. After 3 years of treatment, more than 50% of patients showed improvement in nasal symptoms compared to the placebo group. According to Malling's criteria, these findings support the value of this treatment method in seniors [28].

A second paper documents a similar degree of efficacy and safety for SLIT treatment over the course of three years in seniors over 60 with intermittent, moderate, or severe allergic rhinitis caused by allergies to grass pollen. The efficacy of this treatment, as measured by a reduction in nasal symptoms during the pollen season, was over 55% compared to the placebo. Additionally, three years of SLIT treatment led to a 68% reduction in the use of symptomatic drugs during the pollen season compared to the placebo group [29]. Both studies confirmed a favourable safety profile for AIT treatment.

The first study to document the safety of injection allergen immunotherapy (SCIT) for grass and tree pollen in a large number of patients compared 116 seniors to a control group of 139 young people undergoing the same treatment. In this study, 348 (6.4%) local reactions up to 10 cm in diameter were recorded. There were no systemic reactions. Among 5521 injections performed in young patients, the study recorded 459 local reactions (8.3%) and two cases of systemic reactions of generalized skin pruritus and abortive symptoms of rhinitis or conjunctival pruritus that subsided within an hour after vaccination. No other adverse reactions were observed. The limitation of this report was the lack of healthy control and placebo treated groups [30].

In recent years, only a single randomized and placebo-controlled study confirmed the efficacy and safety of SCIT in elderly patients. A total of 58 elderly patients with allergic rhinitis who were monosensitized to HDM were randomized to receive 2 years of perennial SCIT using PURETHAL Mites or a placebo. The symptoms, medication scores, quality of life, nasal allergen provocation responsiveness and serum allergen-specific IgG4 to D. pteronyssinus and D. farinae and Der p1 and Der p2 were monitored [31]. After 24 months of SCIT, clinical parameters showed significantly decreased serum specific IgG4 against D. pteronyssinus and D. farinae, while Der p1 and Der p2 increased in the treatment group. Two mild systemic anaphylactic reactions: urticaria or erythema with pruritus were reported after SCIT in the treatment group. This study showed that SCIT treatment for HDM allergens was effective and safe in elderly patients with allergic rhinitis [31].

## THE LONG-TERM EFFECT AFTER ALLERGEN IMMUNOTHERAPY IN SENIORS

The long-term effects of AIT treatment in seniors must be considered in the context of the ageing immune system and multimorbidity.

A sustained positive effect was observed over the course of a 3-year period following discontinuation of SCIT for grass pollen allergy in 66.2 ± 2.7-year-old elderly patients with allergic rhinitis [32]. In brief, 34 elderly patients who received preseasonally injected SCIT or placebo for grass pollen allergy were monitored for three years and compared with a placebo group. The combined symptom medication score (CSMS - reflects the sum of daily Symptom Score and daily Medication Score (dMS) comprising the rating of 6 symptoms and use of symptomatic medication), serum level of IgG4 to Phleum pratense and quality of life were assessed immediately after AIT and again 3 years later [32]. Immediately following AIT, the CSMS significantly decreased from 2.15 to 1.13 (*P* = 0.03) and remained lower (1.41 vs. 2.41) than the placebo group during the three years follow-up period after SCIT discontinuation. Serum-specific IgG4 antibodies increased during AIT treatment and remained high at the follow-up observation. Based on the Rhinoconjunctivitis Quality of Life Questionnaire, quality of life was significantly increased specifically in patients who received SCIT, and this improvement was sustained for three years after AIT discontinuation. This study provided the first evidence for long-term efficacy of AIT treatment in the older population [32].

Additionally, an ongoing study of the long-term effects of SLIT on allergies to grasses and mites showed sustained efficacy up to 5 years following discontinuation of AIT treatment [33,34]. These observations also confirmed an increase in IgG4 titer for desensitized allergens, which demonstrates the flexibility of the immune system in old age. The latest study is a 7-year follow-up of 56 seniors who, at the time of completion of SLIT-HDM, had a confirmed effectiveness in terms of clinical symptoms and drug reduction in perennial allergic rhinitis symptoms medication score (SMS) from 4.32 to 1.66. After another seven years, SMS are needed to investigate the effect of allergic rhinitis (AR treatments, particularly AIT. The EAACI defined the CSMS as the harmonized standard for the primary endpoints for AIT trials.) remained at 2.28 vs. 4.12 for the similar placebo group. During this time, the reduction in prescriptions for symptomatic medications varied by 32% in favour of desensitised patients. There was a lower risk of developing new asthma in the desensitised group: 8% vs. 20%. Unfortunately, in the last 5 years, other studies have been lacking on this age group [35^▪▪^].

## SAFETY, COMORBIDITIES, AND ALLERGEN IMMUNOTHERAPY

The safety of AIT was analysed in most of the presented studies. However, detailed data on local and systemic reactions are often imprecise due to a lack of information on the rates of incidence. In most of the prior studies, no systemic reactions were observed, while those reactions that did occur were single, mild events of urticaria or rash. One study of SCIT treatment observed local adverse reactions in only 1.43% of the treatment group [28].

In most of the presented studies, the elderly population had comorbidities that were not contraindications to AIT (including stable ischaemic heart disease, type II diabetes, and hypertension). These diseases did not significantly affect the efficacy and safety of AIT treatment in elderly patients compared to younger patients. Currently, a multidrug regimen is common in the population over 65 years of age. For example, antihypertensive drugs are among the most frequent medications taken. In everyday practice, allergists often have trouble in prescribing treatment to patients who take β-blockers and ACE inhibitors [20,21]. However, contraindications for AIT treatment are the same for all age groups. β-blockers and ACE inhibitors are contraindicated only for SCIT and not SLIT.

Based on these data, the EAACI recommends AR-induced desensitization for patients over 60 years of age if symptomatic drugs prove ineffective [13]. It is possible that the use of epinephrine in elderly patients, particularly those with cardiovascular disease, may place patients at greater risk for drug side effects. However, the data suggest that these complications may be manageable [36^▪^,^37^].

## UNMET NEEDS

There is an unmet need for case studies and multicentre clinical trials on large groups of patients to confirm the efficacy and safety of AIT treatment in seniors. There are also no studies evaluating the effect of AIT on better control of allergic asthma in seniors, for example, hypersensitivity to HDM. There is a particular need to individualize such treatments in seniors. Developing criteria for treatment presents another crucial problem.

Key challenges in developing criteria for AIT treatment include determining the correct endotype of chronic rhinitis and excluding vasomotor and senile rhinitis. The decision to start AIT must be unequivocal and supported by clinical evidence of IgE-dependent allergy. This requires accurate diagnosis and observation of the effects of symptomatic treatment.

## CONCLUSIONS

AIT may be beneficial for the treatment of seniors with respiratory allergic disease, especially those with allergic rhinitis and confirmation of clinically relevant IgE-mediated sensitization to common allergens. SCIT or SLIT may also reduce the need for medication and improve symptom scores in the long term.

## Acknowledgements

*None.*

### Financial support and sponsorship

*None.*

### Conflicts of interest

*There are no conflicts of interest.*

## REFERENCES AND RECOMMENDED READING

Papers of particular interest, published within the annual period of review, have been highlighted as:

▪ of special interest
▪▪ of outstanding interest
